# The Influence of Tractor-Seat Height above the Ground on Lateral Vibrations

**DOI:** 10.3390/s141019713

**Published:** 2014-10-22

**Authors:** Jaime Gomez-Gil, Francisco Javier Gomez-Gil, Rebeca Martin-de-Leon

**Affiliations:** 1 Department of Signal Theory, Communications and Telematics Engineering, University of Valladolid, Valladolid 47011, Spain; E-Mail: rmarleo@ribera.tel.uva.es; 2 Department of Electromechanical Engineering, University of Burgos, Burgos 09006, Spain; E-Mail: fjggil@ubu.es

**Keywords:** tractor, vibration, whole-body vibration, comfort, shock, dose, suspension, terrain, health, low-back pain, cab, agriculture

## Abstract

Farmers experience whole-body vibrations when they drive tractors. Among the various factors that influence the vibrations to which the driver is exposed are terrain roughness, tractor speed, tire type and pressure, rear axle width, and tractor seat height above the ground. In this paper the influence of tractor seat height above the ground on the lateral vibrations to which the tractor driver is exposed is studied by means of a geometrical and an experimental analysis. Both analyses show that: (i) lateral vibrations experienced by a tractor driver increase linearly with tractor-seat height above the ground; (ii) lateral vibrations to which the tractor driver is exposed can equal or exceed vertical vibrations; (iii) in medium-size tractors, a feasible 30 cm reduction in the height of the tractor seat, which represents only 15% of its current height, will reduce the lateral vibrations by around 20%; and (iv) vertical vibrations are scarcely influenced by tractor-seat height above the ground. The results suggest that manufacturers could increase the comfort of tractors by lowering tractor-seat height above the ground, which will reduce lateral vibrations.

## 1. Introduction

The operators of agricultural tractors are exposed to vibrations of different types and intensities. For instance, they experience Whole Body Vibrations (WBV) when driving a farm machine over uneven terrain. The frequency and direction of the vibrations are very important when assessing the effects of WBV on the operator, which are fourfold: degraded health, impaired activities, impaired comfort, and motion sickness [1,2]. Medical studies have clearly demostrated that daily exposure to vibrations will impair the health of the driver [3,4]. Excessive exposure to WBV and awkward working postures are considered to be the major stressors contributing to the development of musculoskeletal complaints among professional drivers [5].

Modern tractors are often equipped with components to reduce vibrations, among others: (i) the tires, especially low-pressure modern tires, as the lower the tire pressure the weaker the tractor vibrations [1,6–8]; (ii) the seat suspension system, certainly, the most popular and effective solution [8–10]; (iii) the front-axle suspension, which can reduce vibrations by as much as 30% [11,12]; and; (iv) the cab suspension system, which along with other tractor components contributes to tractor comfort [8,13,14]. Suspension systems can be conventional, based on coil or air springs together with hydraulic dampers, or can be semi-active or active, which incorporate electronic sensors and continuously adapt some suspension parameters to reduce vibrations more effectively [15–20]. Nevertheless, despite these advances, Scarlett *et al.* [21] found in their experiments with modern, state-of-the-art agricultural tractors, that the limits stipulated in recent legislation [22] on exposure levels to WBV were in excess of 5% and 27% of their field vibration analysis, when the analysis was conducted over periods of 8 and 15 h, respectively.

Our aim is to study the influence of tractor-seat height above the ground on the lateral and vertical vibrations received by the tractor driver. We assumed that the tractor seat and cabin suspensions do not decrease lateral vibrations. We hypothesize that lowering tractor-seat height will reduce lateral vibrations.

## 2. Theoretical Considerations

**Figure 1.**
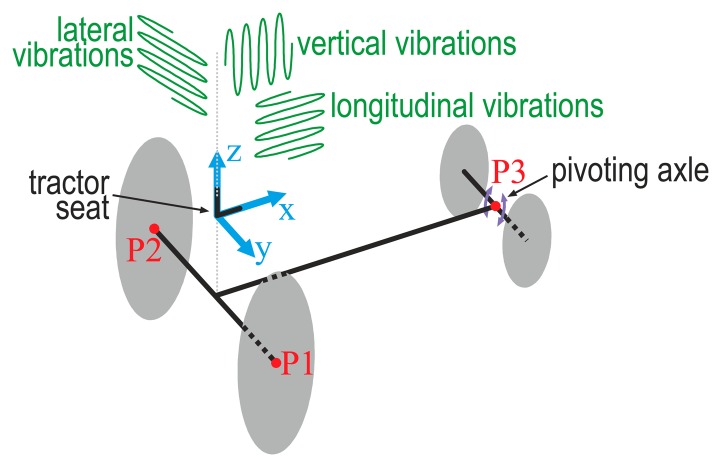
Scheme of a tractor that shows the coordinate axis used in this study (blue), the three points P1, P2 and P3 at which the chassis is attached to the tractor (red), the rotation capacity of the front axle (violet), and the spatial movement of the lateral, vertical and longitudinal vibrations (green).

Some basic considerations about vertical, lateral and longitudinal vibrations need to be made, for a better understanding of the research results. Assuming that the coordinate axes are positioned over the tractor, as outlined in Figure 1, the vertical vibrations are those that occur on the *Z* axis, the lateral vibrations are those that occur on the *Y* axis, and the longitudinal vibrations are those that occur on the *X* axis.

Although a tractor has four wheels, it follows the tricycle model that is characterized by having two rear wheels and one frontal steering wheel. The chassis of a tractor extends over points P1, P2 and P3 shown in Figure 1. Point P3 is in the middle of the front axle. This axle rotates within the YZ plane with its centre of rotation at P3. In this geometry: (i) lateral vibrations are produced when a rear wheel moves over uneven terrain and the opposite rear wheel moves over level terrain; and (ii) vibrations on the front axle will not influence the lateral vibrations. The vertical movements in the middle of the front axle, at point P3, will mainly produce a pitch motion and a change in the vertical movements of the tractor.

**Figure 2.**
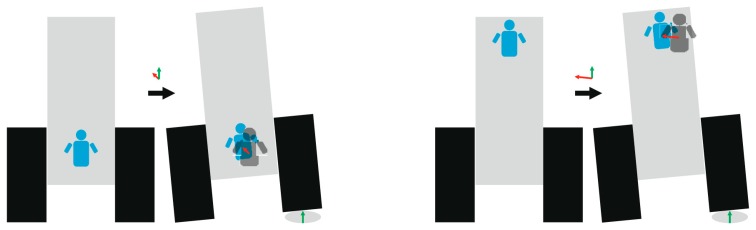
Diagram of a rear view of the tractor depicting the vibrations transmitted by uneven terrain when the tractor seat is in a low position (**left**) and when the tractor seat is in a high position (**right**). The red arrow represents the displacement of the driver caused by the displacement of one wheel of the tractor (green arrow), moving over uneven terrain. Note that the red arrow is smaller in the figure on the left than in the figure on the right.

Tractor-seat height above the ground is one of the factors that influence the magnitude and the type of vibrations received by the driver. Figure 2 depicts this influence in a simplified model, in which we consider two situations. In the first situation, seat height over the ground is low and uneven terrain produces vibrations in the tractor that are transmitted to the driver, mainly in the form of vertical vibrations. The second is when tractor-seat height over the ground is high and uneven terrain produces vibrations that are transmitted to the driver mainly in the form of vertical as well as lateral vibrations.

## 3. Methods

### 3.1. Materials

A New Holland TM-190 tractor was modeled in a geometrical study. The tractor was equipped with vibration acquisition hardware and was subjected to real tests in this experimental study.

The tractor was equipped with VF710/60R42 Michelin Xeobib tires on the rear axle and 540/65R30 Good Year Optitrac DT818 tires on the front axle. The front and rear axle tires were inflated at 150 and 100 kPa, respectively, which are the recommended pressures for this tractor. The height of the seat above the ground, H, was 190 cm, and the end-to-end distance between the wheels of the rear axle, T, was 287 cm. This tractor came equipped with the terraglide front-axle suspension system and with the comfort ride cabin suspension system.

**Figure 3.**
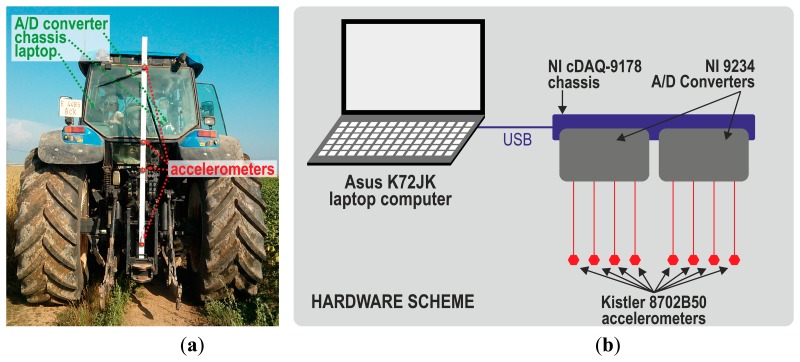
(**a**) Tractor employed. It had a rectangular metal tube screwed to it, and eight accelerometers were attached to the tube at four different heights; (**b**) Hardware scheme of the data acquisition system.

Eight Kistler 8702B50 piezoelectric accelerometers were employed. Their key specifications were an acceleration range of ±50 g, a sensitivity of 100 mV/g, and a frequency range of 0.5–10,000 Hz. Accelerometers were connected to two National Instruments 9234 four-channel A/D converters with anti-aliasing filters, the key specifications of which are 51.2 kS/s and 24 resolution bits. These converters were inserted into a National Instruments cDAQ-9178 chassis, which was connected to an Asus k72jk notebook with a Microsoft Windows 7 Home Premium 64-bit operating system. Figure 3 presents the placement and connection of the elements employed in the real tests of this study.

### 3.2. Procedure

Two studies, one geometrical and the other experimental, were conducted on the influence of tractor-seat height on vibrations.

#### 3.2.1. Geometrical Study of the Influence of Tractor-Seat Height on Vibrations

**Figure 4.**
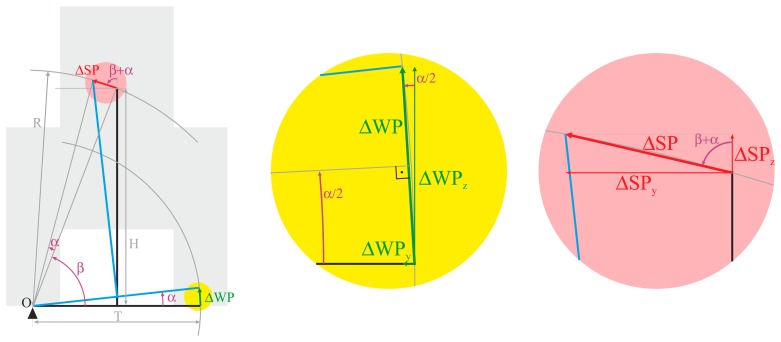
Geometrical relation between tractor seat movement ΔSP over the YZ plane and displacement of the rear tractor wheel ΔWP. Graphs represent the rear view of the tractor.

The way in which the irregularities of the terrain are transmitted to the driver in relation to the height of the tractor seat above the ground can be obtained by geometrical methods. Figure 4 represents the tractor movement produced by an irregularity in the ground, considering the tractor as a rigid body with no inertia. In this figure, **O** is the tractor rotation center in the movement due to the rear wheel moving over uneven terrain, a point which coincides with the fulcrum of the opposite rear wheel, **T** is the tractor rear axle width, **H** is the height of the tractor seat with reference to the ground, **β** is a parameter that depends on T and H, **α** is the angle of the rear axis to the ground produced by unevenness on the ground that displaces **ΔWP**, the Wheel Position of one of the wheels, and **ΔSP** is the same displacement measured on the Seat Position.

A ΔWP_z_ vertical displacement of one tractor wheel produces two displacements of the tractor seat, a vertical one on the *Z* axis, and another lateral one on the *Y* axis. Taking into account the parameters presented in Figure 4 and the trigonometric functions, the vertical and lateral displacement of the tractor seat, ΔSP_z_ and ΔSP_y_, can be computed as a function of the vertical displacement of one tractor wheel, ΔWP_z_, as follows:

(1) $$\sin\left(\beta +\alpha \right)=\frac{H+\Delta SP_{z}}{R}\;\Rightarrow \;\Delta SP_{z}=R\cdot sin\left(\beta +\alpha \right)-H$$

(2) $$\cos\left(\beta +\alpha \right)=\frac{\overset{T}{\;}/\underset{2}{\;}-\Delta SP_{y}}{R}\;\Rightarrow \;SP_{y}=\frac{T}{2}-R\cdot cos\left(\beta +\alpha \right)$$

in which:

(3) $$R=\sqrt{H^{2}+{\left(T/2\right)}^{2}}$$

(4) $$\beta =\text{arctan}\;\left(\frac{H}{X/2}\right)$$

(5) $$\alpha =2\cdot \text{arcsin}\;\left(\frac{\Delta WP}{2\cdot T}\right)\;\text{with}\;\Delta WP=\frac{\Delta WP_{z}}{cos\left(\alpha /2\right)}$$

A transmission factor over on the *z* axis (*tf_z_*) and a transmission factor over the *y* axis (*tf_z_*) were defined as the ratio between the displacement on an axis of the tractor seat (*ΔSPz* and *ΔSPz*, respectively), and the elevation of a tractor wheel (*ΔWPz*):

(6) $$tf_{z}=\frac{\Delta SP_{z}}{\Delta WP_{z}}$$

(7) $$tf_{y}=\frac{\Delta SP_{y}}{\Delta WP_{z}}$$

#### 3.2.2. Experimental Study of the Influence of Tractor-Seat Height on Vibrations

A rectangular metal tube was screwed vertically into the rear of the tractor (Figure 3a). Four attachments to the tractor chassis were set up, in order to minimize resonance vibrations. The tube was very near the rear axle and was assumed to be in the middle of the rear axle, in order to simplify data preprocessing. Four accelerometers where attached to the rear side of the metal tube at heights of 0.8, 1.6, 1.9, and 2.7 m above the ground, and another four in the left side at the same heights. The measurements acquired with accelerometers attached to this tube were not influenced by the cabin suspension system, because the tube was screwed to the chassis of the tractor.

**Figure 5.**
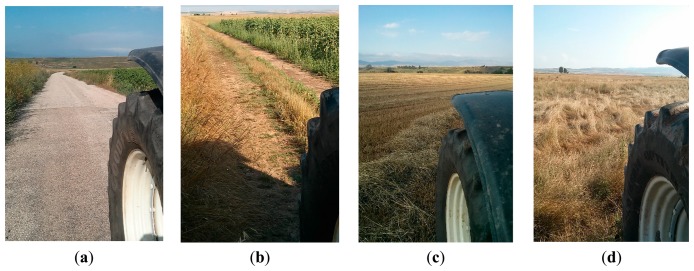
Test surfaces: (**a**) paved road; (**b**) unpaved road; (**c**) cultivated plot; and (**d**) uncultivated plot.

Four sets of vibration data were acquired in Aguilar de Bureba, Burgos, Spain. The first one was acquired on a paved road (Figure 5a), the second, on an unpaved road (Figure 5b), the third on a cultivated plot (Figure 5c), and the forth on an uncultivated plot (Figure 5d). On each type of ground, acceleration signals were acquired for five minutes with the eight accelerometers at a rate of 5000 samples per second. The tractor moved over the four surfaces at a constant speed of 8 km/h.

The power spectrum density in the 1–2500 Hz band and the mean acceleration of the vibrations falling within the range 1–7 Hz were computed for each of the 32 temporal vibration signals acquired.

The power-spectrum densities were computed using periodogram averaging techniques [23]. To do so, the periodogram Matlab command was applied over eighteen five-second windows for each acceleration signal, and the average of eighteen power spectrum densities was computed.

The mean accelerations were computed in the following steps: (i) the average power spectrum density in the 1–7 Hz frequency-(m/s^2^)^2−^ was computed from the power spectrum density-(m/s^2^)^2^/Hz-; and; (ii) the square root of the average power spectrum-(m/s^2^)-was calculated.

## 4. Results

In this section, the results of both the geometrical study and the experimental study of the influence of tractor-seat height on vibrations are presented and compared.

### 4.1. Results of the Geometrical Study of the Influence of Tractor-Seat Height on Vibrations

**Figure 6.**
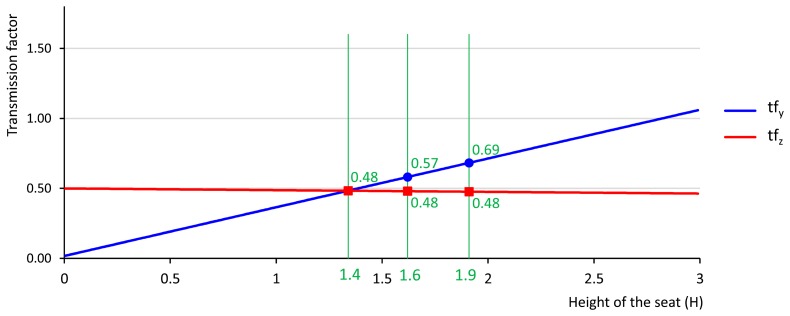
Transmission factor on the *z* and the *y*-axis (tf_y_ and tf_y_) of the graph as a function of tractor-seat height above the ground. The graph was computed for a Wheel Position vertical displacement of ΔWP_z_ = 0.1 m and for a tractor with an end-to-end rear-axle width of 2.87 m. The tractor-seat height of 1.9 m above the ground in this test is represented by a vertical line, and the transmission factor on the *z* and the *y*-axis, 0.48 and 0.66, respectively, are labeled on this line. Another vertical line represents the tractor-seat height at 1.4 m, a height at which the transmission factors on the *z* and *y*-axis are equalized, the value of which is 0.48.

A plot of the transmission factors, geometrically computed in Section 4.1 as a function of tractor seat height above the ground, is shown in Figure 6. In this figure:

- The elevation of a tractor wheel by a single unit of length is transmitted to the tractor seat in 0.5 length units, if the tractor seat is above the ground (H = 0). The 0.5 factor reflects the position of the seat, assumed to be in the middle of the rear tractor axle.
- The vertical vibrations of the tractor seat decrease, but only very slightly, when the height of the tractor-seat height above the ground increases.
- The lateral vibrations on the Y axis are 0, if the tractor seat is above the ground, and they increase linearly with H.
- A driver of a tractor with an end-to-end rear-axle width of 2.87 m, seated at a height of 1.9 m above the ground, as is the case of the tractor tested in this article, will suffer more lateral vibrations than vertical vibrations.
- A driver of a tractor with an end-to-end rear-axle width of 2.87 m, as in the case of the tractor tested in this article, will experience similar lateral and vertical vibrations, if the tractor seat is placed at a height of 1.4 m above the ground.

### 4.2. Results of the Experimental Study of the Influence of Tractor-Seat Height on Vibrations

**Figure 7.**
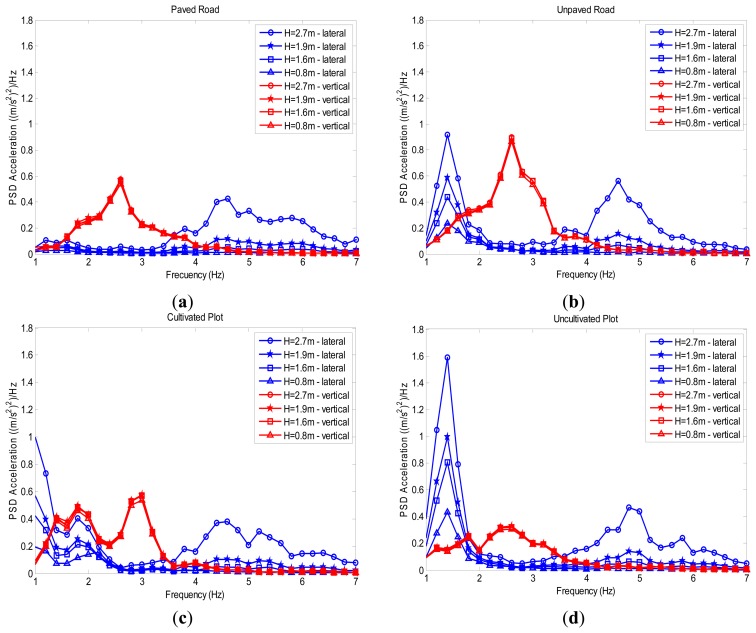
Power spectrum density plots of measurements acquired with accelerometers placed on the rear axle of the tractor at heights of 0.8, 1.6, 1.9, and 2.7 m above the ground. Graphs are shown for vertical (red) and lateral (blue) vibrations on paths along the paved road (**a**), the unpaved road (**b**); the cultivated plot (**c**); and the uncultivated plot (**d**).

The power spectral densities of the vibrations acquired are presented in Figure 7. Eight power spectral densities are plotted for each type of surface, four of which correspond to vertical accelerations at 0.8, 1.6, 1.9, and 2.7 m, and four of which correspond to lateral vibrations at the same heights. It can be perceived from these plots that: (i) the vertical vibrations are scarcely influenced by the sensor height above the ground; while (ii) the power spectrum density of the lateral vibrations significantly increases at this height.

The mean accelerations of each of the 32 signals, acquired by taking into account only the vibrations falling in the 1–7 Hz frequency band, are presented on Table 1.

**Figure 8.**
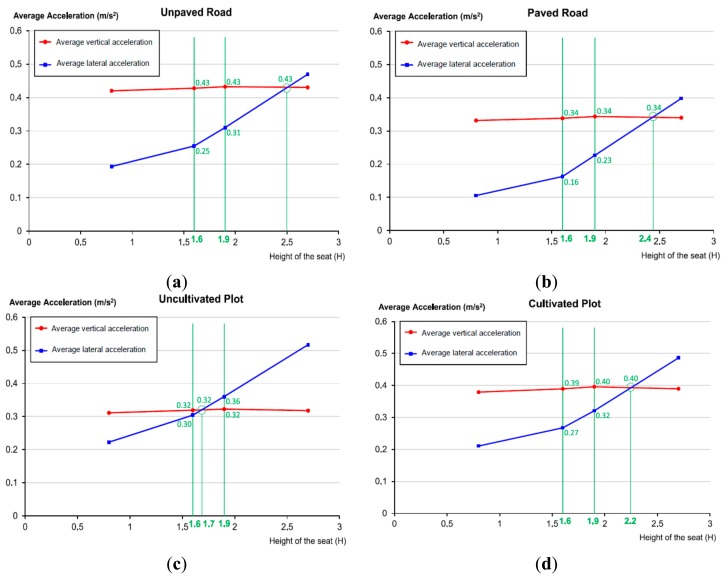
Transmission factors on the *z* and the *y*-axis geometrically computed in Section 3 (green color) *versus* mean acceleration on the 1–7 Hz frequency band (red color) obtained experimentally on paths along the paved road (**a**); the unpaved road (**b**); the cultivated plot (**c**); and the uncultivated plot (**d**) for the four tractor-seat heights (0.8, 1.6, 1.9, and 2.7).

Figure 8 is presented so that we can graphically perceive the evolution of the mean acceleration of the vibrations falling in the 1–7 Hz frequency band in relation to tractor-seat height above the ground. The graphs in this figure are generated by using data from Table 1. According to Figure 8:

- The mean vertical acceleration of the vibrations falling in the 1–7 Hz frequency band of the tractor seat are slightly influenced by the height of the tractor-seat above the ground.
- The mean lateral acceleration of the vibrations falling in the 1–7 Hz frequency band of the tractor seat increase along with the height of the tractor seat above the ground.
- The 2.4 m value for the paved road, the 2.5 m value for the unpaved road, the 2.2 m value for the cultivated plot, and the 1.7 m. value for the uncultivated plot are threshold values. At these heights, mean acceleration in the 1–7 Hz frequency band is the same for both the vertical and the lateral vibration components. Below these heights, the vertical vibration component is greater than the lateral vibration component. And above these heights, the lateral vibration component is higher than the vertical vibration component.

### 4.3. Comparison of Geometrical and Experimental Studies of the Influence of Tractor-Seat Height on Vibrations

The results of the geometrical and the experimental studies match each other, in so far as they both show that:

- Tractor-seat height above the ground slightly influences the vertical vibrations experienced by the tractor driver; vertical vibrations remain almost unaffected by seat-height above the ground.
- Tractor-seat height above the ground notably influences the lateral vibrations experienced by the tractor driver; lateral vibrations are highly dependent on seat-height above the ground.
- Lateral vibrations increase along with the height of the tractor seat above the ground, and there is a specific height of the tractor-seat above the ground in accordance with the type of terrain that determines the predominant vibration component. Below that height the vertical vibration component is higher than the lateral vibration component, and above that height the lateral vibration component is higher than the vertical vibration component.

### 4.4. The Effect of a 15% Reduction in Tractor-Seat Height above the Ground on Vibrations

The evolution of the analytically computed transmission factors (tf_z_, tf_y_) of the tractor employed in the real test, when the height of the tractor seat is reduced by 15%, from 1.9 to 1.6 m, is presented as a percentage in Table 2.

The evolution of the experimental vertical and lateral mean accelerations in the 1–7 Hz frequency band for the four test surfaces, when the measurement point is reduced by 15%, from 1.9 to 1.6 m, is also presented as a percentage in Table 3.

## 5. Discussion

The main findings of the present study are fourfold: (i) lateral vibrations experienced by a tractor driver increase linearly with tractor-seat height above the ground; (ii) lateral vibrations to which the tractor driver is exposed can equal or exceed vertical vibrations; (iii) in medium-size tractors, a feasible 30 cm reduction in the height of the tractor seat, which represents only 15% of its current height, will reduce the lateral vibrations by around 20%, and it barely influences the vertical vibration; and (iv) vertical vibrations are scarcely influenced by tractor-seat height above the ground. The first finding, that the lateral vibrations experienced by a tractor driver increase linearly with the height of the tractor-seat above the ground, is supported in this article by three facts:

- The diagram in Figure 2 leads us to think that lateral vibrations increase with the height of the tractor-seat above the ground.
- The geometrical study described in Section 3.2.1 proves that the lateral vibrations to which a tractor driver is exposed increase linearly with height, if we consider the tractor as a rigid solid without inertia, because the transmission factor over the *y*-axis (tf_y_), displayed as a red line on Figure 6, increases linearly with H, the height of the tractor-seat above the ground.
- The field test results of Section 3.2.2, and especially the blue lines of the four graphs in Figure 7, show a growing trend in the mean acceleration in the 1–7 Hz frequency band of lateral vibrations in relation to H, the height of the tractor-seat above the ground. This increase can be considered constant, because the blue lines on the four graphs in Figure 8 are almost a straight line.

The second finding, that lateral vibrations experienced by the tractor driver can be as high or even higher than vertical vibrations, is supported in this article by two facts:

- The geometrical study of the Sections 3.2.1 and 4.1 proves that, if we consider the tractor as a rigid solid with no inertia, the driver of a tractor with a 2.87 end-to-end rear-axle width will experience more lateral vibration than vertical vibration, if the tractor seat height above the ground is over 1.4 m.
- The field test results of Section 4.2, and specially the four graphs of Figure 8, show that average lateral acceleration is higher than average vertical acceleration at heights above the ground of over 2.4, 2.5, 2.2, and 1.6 m, respectively for the four surfaces that were tested.

The first of the third finding, that a 15% reduction in the height of the tractor-seat above the ground can reduce the lateral vibrations of the tractor by 20%, is supported in this article by two facts:

- According to data from Table 2, the analytically computed transmission factor on the y-axis (tf_y_) for the tractor employed in the real tests is reduced by 21%, when the height of the tractor seat is reduced from 1.9 to 1.6 m. A 21% percentage is close to a 20% percentage.
- According to data from Table 3, the experimental mean acceleration in the 1–7 Hz frequency band, on the *y*-axis, decreased with a 15% reduction in the height of the tractor-seat, on all four test surfaces, by 39%, 21%, 20%, and 18%. These four percentages respectively refer to the paved, unpaved, cultivated, and uncultivated surfaces and are close to or higher than 20%.

The second of the third finding, that a 15% reduction in the height of tractor seat above the ground will not influence the vertical vibrations of the tractor, is supported in this article by two facts:

- According to data from Table 2, the analytically computed transmission factor on the z-axis (tf_z_) for the tractor employed in the real tests increased by 1% when the height of the tractor seat above the ground decreased from 1.9 to 1.6 m. A 1% percentage is negligible.
- According to data from Table 3, the experimental mean acceleration in the 1–7 Hz frequency band, on the *z*-axis, decreased with a 15% reduction in the height of the tractor seat on all four test surfaces by 2%, 1%, 2%, and 1%. These four percentages respectively refer to the paved, unpaved, cultivated, and uncultivated surfaces, and are negligible.

The four finding, that vertical vibrations are scarcely influenced by the height of the tractor-seat above the ground, is supported in this article by three facts:

- The diagram in Figure 2 leads us to think that vertical vibrations are not influenced by the height of the tractor-seat above the ground.
- The geometrical study of Section 3.2.1 proves that, if we consider the tractor as a rigid solid with no inertia, the vertical vibrations that a tractor driver experiences undergo a slight linear decrease with the height of the seat, because the transmission factor over the *z*-axis (tf_z_), displayed as a red line on Figure 6, slightly decreases linearly with H, the height of the tractor-seat above the ground.
- The field test results of Section 4.2, and especially the red lines of the four graphs in Figure 7, show that the mean acceleration in the 1–7 Hz frequency band of lateral vibrations in relation to H, the height of the tractor seat above the ground, are slightly modified. This slight experimental increase is almost a straight line with a zero slope, as shown by the red lines in Figure 8.

The previous conclusions are also valid for a real tractor with seat suspension and/or with cabin suspension.

- This study concludes that lateral vibrations increase linearly with tractor seat height above the ground for tractors that neither have a cabin nor seat suspension:
  ○ Most tractor seats and cabins only reduce vertical and longitudinal vibrations, but not lateral vibrations. Thus, the lateral vibrations for this kind of tractor will increase linearly with tractor seat height above the ground, as this study has found.
  ○ A few tractors are equipped with seats and/or cabins that reduce the lateral vibrations. If we suppose that the suspension decreases the vibrations by a constant percentage, regardless of the amount of vibrations, then the increase of lateral vibrations with height will be also linear. On the basis of this supposition, the conclusions of our study regarding lateral vibrations are valid for this kind of tractor. If this supposition is not upheld, the conclusions of our study regarding lateral vibrations are almost acceptable, because the lateral vibrations will increase with tractor seat above the ground, although this increase may not be linear.
- This study concludes that vertical vibration is scarcely influenced by seat height above the ground when neither seat suspension nor cabin suspension are considered. The height will therefore neither increase nor decrease the vertical vibrations if seat and/or cabin suspension are considered, because the seat and the cabin will act with the same amount of vertical vibration whenever seat height above the ground is modified. Thus, for vertical vibration, our study is completely valid both for tractors with and without seat and/or cabin suspension.

As far as the authors are aware, there are no studies that have quantified the influence of tractor-seat height above the ground on the vibrations experienced by the tractor driver. Nevertheless, in agreement with the results of our study, those of Scarlet *et al.* [21] and Marsili *et al.* [11] show that lateral vibrations in tractors are of similar or even higher magnitudes than vertical vibrations. Seat and cabin suspension is not assumed to decrease lateral vibrations. The following line of reasoning supports this assumption:

- The seat suspension system [9–11,24] is an important component of the tractor that can reduce vibrations. Nevertheless, most tractors seats only reduce the vertical component of vibrations, because damping lateral and longitudinal oscillations of the seat could negatively interfere in safe tractor driving. As far as the authors know, the two main companies that manufacture seats for tractors are *Grammer* [25] and *Sears seating* [26]. It is a representative fact that of the 29 models that both companies manufactured as agricultural equipment in 2013, only 6 of them incorporated a simple lateral suspension system. It may therefore be said that tractor seats are components that only reduce vertical and longitudinal vibrations.
- Tractor cab suspension [8,13,14] systems are becoming popular in agricultural tractors. A tractor cab suspension system that only reduces vertical vibrations is simple and economic, because it is usually based on springs or air springs and on viscous dampers. These tractor cabs are usually equipped with Panhard rods that prevent lateral movement. A tractor cab suspension system that also reduces lateral vibrations is more complex, because it needs active systems to cancel the lateral accelerations produced by tractor turns at high speed. As far as the authors know, only the three-point tractor cab suspension systems in the Fendt 900 series and some Renault (now Class) tractors [27] have an effective lateral suspension. Considering that only a single series of tractors from each of the two companies are equipped with cabs that reduce lateral vibrations, it is reasonable to consider that tractor cab suspension systems do not usually reduce lateral vibrations.

In addition to seat and cabin suspension systems, tractors are equipped with tires [15,16] and sometimes with a front-axle suspension system [11,12]. An analysis is presented below of the influence of these four elements, with respect to lateral vibrations to which the tractor driver is exposed:

- The tires, especially low-pressure tires, probably reduce tractor vibrations more than any other component. Besides, low-pressure tires reduce vibrations in all of their components: vertical, longitudinal and lateral vibrations. Nevertheless, taking into account firstly that the geometrical study of this work has been done without consider the effect of the tires, tacking in account secondly that the real experiments have been carried out with a tractor equipped with tires, and taking into account finally that both, the geometrical and the experimental studies, match fine in the found findings, the conclusions of this study will be valid for a tractor equipped with tires.
- The tractor chassis is joined to the tractor at three points: at the left end of the rear axle, at the right end of the rear axle, and at the midpoint of the pivoting front axle. Due to this pivot feature of the front axle, the passage of the front wheels over uneven terrain will not expose the driver to lateral vibrations. The front-axle suspension system of a tractor helps to ensure stable driving and to reduce the pitch, but it has little influence on the amount of lateral vibrations experienced by the tractor driver.

A synthesis of the previous analyses reveals that only the low pressure tractor tire wheels help to reduce lateral vibrations, while the seat suspension system, the front-axle suspension system, and the tractor cab suspension system are not usually designed to reduce the lateral vibrations experienced by the tractor driver or do so only very slightly. This conclusion reinforces the need for acceptable tractor geometries, to avoid excessive lateral vibrations, and for tractor-seat height above ground to be reduced, the importance of which is underlined in this study on the reduction of lateral vibrations.

**Figure 9.**
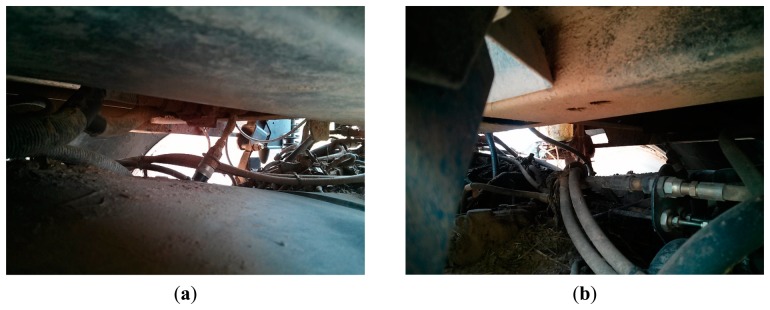
Photos of the space under the cab of the New Holland TM190 tractor tested in this article. These photos show that a reduction in tractor-seat height could be feasible and easy to achieve for tractor manufacturers.

The tractor geometry design implies a trade-off between driver visibility, constructability, weight distribution, pulling capacity, and aesthetic considerations among others tractor features. A large reduction of the height of the tractor-seat above the ground might reduce driver visibility and will complicate the design process of the tractor. However, it is the authors' opinion that a reduction of 30 cm, for example, in medium-size tractors, equal to 15%, is feasible, easy for tractor manufacturers to achieve, and would hardly effect driver visibility. Figure 9 show two photos of the space under the cab of the tractor tested in this article. The presence of a lot of space in these photos suggests that a 30 cm reduction of tractor-seat height above the ground could be feasible and simple to achieve.

Exposure to lateral vibrations is more harmful than exposure to vertical vibrations. This is reflected by the coefficient applied to the frequency weighting acceleration values for lateral and vertical vibrations in the ISO Standard 2631 [28]: *Mechanical vibration and shock-evaluation of human exposure to whole-body vibration*. The multiplication factors applied to vertical vibrations in this standard is 1, while the weighted coefficient applied to lateral vibrations is 1.4, which would suggest that lateral vibrations are about 40% more harmful than vertical ones. This reinforces the need to reduce lateral vibrations.

The New Holland TM-190 tractor employed in the field test described in this article is a popular and representative model for medium and medium-high horsepower tractors. In fact, this model was named tractor of the year 2003 in Europe. Medium-high horsepower tractors, such as for example in the 2013 year market, those of the 6R, 7R and 8R series from the John Deere Company, those of the T6, T7 and T8 from the Case New Holland company, and those of the 6400, 7400, 7600 and 8600 series from the Massey Ferguson company are of similar dimensions, so the conclusions of this article are equally applicable to them. There are small tractors on the market, such as vineyard tractors. According to Section 3 and Figure 6, the lateral vibration to which drivers of this tractor type are exposed will also be significant, because both, the rear-axle width of this type of tractor and the height of the tractor seat above the ground, are smaller. So, the conclusion of this article is applicable to most models of tractor on the market.

One strength of the current study is noteworthy: as far as the authors are aware, it is the first research that has shown an increasing linear relation between the height of the tractor-seat above the ground and the lateral vibrations experienced by the tractor driver.

## 6. Conclusions

This study concludes that: (i) doses of lateral vibrations experienced by a tractor driver increase linearly with the height of the tractor seat above the ground; (ii) lateral vibrations to which the tractor driver is exposed can equal or exceed vertical vibrations; (iii) in medium-size tractors, a feasible 30 cm reduction in the height of the tractor seat, which represents only 15% of its current height, will reduce the lateral vibrations by around 20%; and (iv) vertical vibrations are scarcely influenced by tractor-seat height above the ground. In summary, the data suggest that lateral vibrations can be easily reduced by lowering the tractor cabin, and then, by decreasing the height of the tractor-seat. Knowledge of these findings will provide valuable information mainly for manufacturers, in order to reduce the physical exposure of tractor drivers to lateral vibrations.

## Figures and Tables

**Table 1.** Mean acceleration of the vibration signals taking into account only the vibrations falling in the 1–7 Hz frequency band for both vertical and lateral vibration in the experimental test performed over a paved road, an unpaved road, a cultivated plot, and an uncultivated plot. Numeric values are expressed in $\left(\frac{m}{s^{2}}\right)$ units.

	**H = 0.8**	**H = 1.6**	**H = 1.9**	**H = 2.7**
Paved road–vertical	0.3315	0.3382	0.3435	0.3399
Paved road–lateral	0.1054	0.1628	0.2265	0.3981
Unpaved road–vertical	0.4200	0.4278	0.4322	0.4302
Unpaved road–lateral	0.1934	0.2548	0.3100	0.4696
Cultivated plot–vertical	0.3792	0.3894	0.3959	0.3896
Cultivated plot–lateral	0.2107	0.2674	0.3211	0.4864
Uncultivated plot–vertical	0.3110	0.3189	0.3223	0.3178
Uncultivated plot–lateral	0.2225	0.3040	0.3603	0.5168
Mean–vertical	0.3604	0.3686	0.3735	0.3694
Mean–lateral	0.1830	0.2472	0.3045	0.4677

**Table 2.** Evolution of the analytical transmission factors (tf_z_, tf_y_) values of the tractor employed in the real test when the height of the tractor seat is reduced by 15%, from 1.9 to 1.6 m.

	**H = 1.9**	**H = 1.6**	**Δ%**
Analytical TFz	0.476	0.479	1%
Analytical TFy	0.696	0.587	−19%

**Table 3.** Evolution of the experimental vertical and lateral mean accelerations in the 1–7 Hz frequency band for the four surfaces in the test, when the measurement point is reduced by 15%, from 1.9 to 1.6 m.

	**H = 1.9**	**H = 1.6**	**Δ%**
Experimental mean vertical acceleration in the 1–7 Hz frequency band on the paved road	0.3435	0.3382	−2%
Experimental mean lateral acceleration in the 1–7 Hz frequency band on the paved road	0.2265	0.1628	−39%
Experimental mean vertical acceleration in the 1–7 Hz frequency band on the unpaved road	0.4322	0.4278	−1%
Experimental mean lateral acceleration in the 1–7 Hz frequency band on the unpaved road	0.31	0.2548	−21%
Experimental mean vertical acceleration in the 1–7 Hz frequency band on the cultivated plot	0.3959	0.3894	−2%
Experimental mean lateral acceleration in the 1–7 Hz frequency band on the cultivated plot	0.3211	0.2674	−20%
Experimental mean vertical acceleration in the 1–7 Hz frequency band on the uncultivated plot	0.3223	0.3189	−1%
Experimental mean lateral acceleration in the 1–7 Hz frequency band on the uncultivated plot	0.3603	0.3040	−18%
